# Conditional survival and the prognostic value of serum carcinoembryonic antigen level in oldest old with colorectal cancer

**DOI:** 10.1186/s12876-024-03318-4

**Published:** 2024-07-10

**Authors:** Weijing He, Yufei Yang, Qi Liu, Dakui Luo, Qingguo Li, Xinxiang Li

**Affiliations:** 1 Department of Colorectal Surgery, Fudan University Shanghai Cancer Center, Xuhui District, #270 Dongan’ Road, Shanghai, 200032 China; 2 Department of Oncology, Shanghai Medical College, Fudan University, Shanghai, China

**Keywords:** Conditional survival, Serum carcinoembryonic antigen, Prognosis, Oldest old, Colorectal cancer

## Abstract

**Background:**

To evaluate the clinical value of serum CEA levels and their implications on the diagnostic value of the conventional TNM staging system in the oldest-old patients with colorectal cancer (CRC).

**Methods:**

The recruited subjects were colorectal cancer patients aged 85 and older. The cutoff value for normal CEA level is 5 ng/mL. Patients with elevated CEA levels were categorized as stage C1, and those with normal CEA levels as stage C0. A number of Cox proportional hazard regression models were established to evaluate the prognosis of different prognostic factors with hazard ratios (HRs) and 95% confidence intervals (CIs). The Kaplan–Meier method was utilized to display the disparate prognostic impact of multiple clinicopathological factors with the log-rank test.

**Results:**

A total of 17,359 oldest-old patients diagnosed with CRC were recruited from the SEER database. The conditional survival of oldest-old patients with CRC was dismal with a 1-year conditional survival of only 11%, 18%, and 30% for patients surviving 1, 3, and 5 years, respectively. Patients with stage C1 exhibited a 48.5% increased risk of CRC-specific mortality compared with stage C0 (HR = 1.485, 95%CI = 1.393–1.583, using stage C0 patients as the reference, *P* < 0.001). All the stage C0 patients indicated lower HRs relative to the corresponding stage C1 patients.

**Conclusions:**

Dismal conditional survival of oldest-old patients with CRC should be given additional consideration. C stage influences the prognosis of oldest-old patients with CRC.

## Background

Colorectal cancer (CRC) is the third most common malignancy worldwide [1]. As the average lifespan increases, the number of elderly patients (≥ 85 years old) with CRC has also increased [2]. Patients aged 85 and above are often considered the oldest-old [3].

In the United States, the median age of diagnosis of colorectal cancer is 67 years, and 11.2% of new cases occur in individuals over the age of 84. The oldest-old category comprises 20.6% of the total deaths caused by CRC [4]. The oldest-old is a specific population in CRC, characterized by multiple comorbidities with increased postoperative morbidity and mortality [5]. Moreover, the Tumor-Node-Metastasis (TNM) staging system developed by the American Joint Committee on Cancer (AJCC) is inadequate for accurately assessing the prognosis of CRC Therefore, there is a need for improvement of the current staging system [6].

Serum carcinoembryonic antigen (CEA) is a highly glycosylated 201 kDa antigen expressed on the apical surface of colon epithelial cells and excreted through the intestinal cavity [7]. CEA is the most widely used blood-based molecular marker for CRC and has been shown to play an important role in monitoring disease progression and predicting disease prognosis [8–11]. The AJCC Colorectal Working Group recommended the inclusion of serum CEA levels (C stage) to complement and modify the anatomic TNM staging of CRC in early 2000.

The present study was a large population-based study designed to evaluate the clinical value of serum CEA levels and their impact on the diagnostic value of the conventional TNM staging system in the oldest-old patients with CRC.

## Methods

### Patients

Using the National Cancer Institute’s Surveillance, Epidemiology, and End Results (SEER) *Stat software, Version 8.3.8 (Surveillance Research Program, www.seer.cancer.gov/seerstat), patients meeting the strict criteria were screened from the SEER database. The latter was an authoritative and comprehensive population-based database containing information on virtually all patients with newly diagnosed malignant tumors, covering approximately 28% of the U.S. population.

**Fig. 1:**
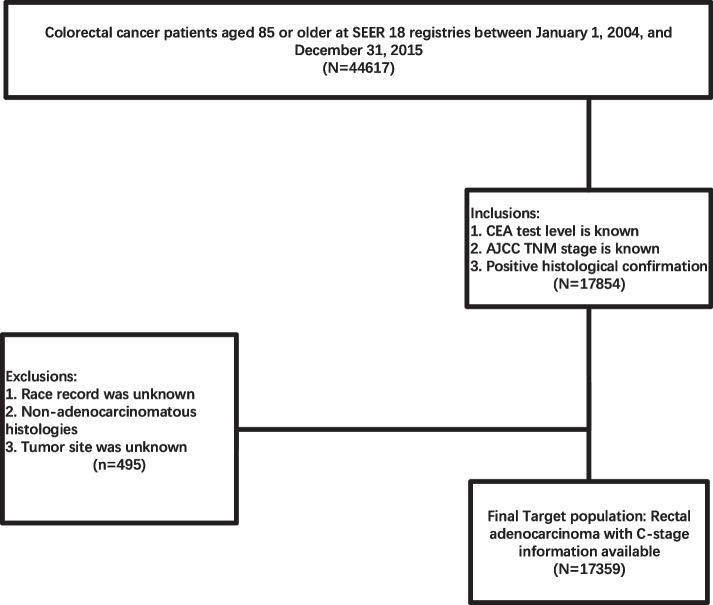
Flow diagram of patient selection

The flow diagram of patient selection was shown in Fig. 1. Initially, CRC patients aged 85 or older were recruited from SEER 18 registries between January 1, 2004 and December 31, 2015. In addition, only CRC patients with known CEA test levels were included in the present study. All cases for analyses were required to have 7th American Joint Committee on Cancer (AJCC) TNM stage and positive histologic confirmation. Patients with unknown race records, non- adenocarcinoma histological profile and unknown tumor site were excluded from our analyses.

### Conditional survival

Conditional survival was defined as the probability of survival and was calculated in the subset of patients who had survived a predefined period. This parameter could therefore provide insight into prognostic prediction and offer better guidance for clinical treatment [12]. This is especially true for the oldest patients, who, due to their poor prognosis, complex functional status, and diminished ability to care for themselves, face important decisions about both their professional and personal lives.. In addition, physicians could perform risk stratification based on conditional survival regarding the frequency and timing of follow-up.

### C–TNM stage

The CEA level of rectal cancer was extracted from the SEER database and assessed before treatment, recorded as “CS Site-Specific Factor 1”. The cutoff value for normal CEA levels is 5 ng/mL. Subsequently, patients with elevated CEA levels were categorized as stage C1, and those with normal CEA levels were categorized as stage C0. In addition each patient with the conventional AJCC TNM stage was assigned C stage, and the C-TNM stage was defined as the combination of the C and the AJCC TNM stages.

### Statistical analysis

In the present study, several Cox proportional hazard regression models were used for evaluation of the impact of different prognostic factors with hazard ratios (HRs) and 95% confidence intervals (CIs). The parameter used in the results of this study was cancer-specific survival (CSS). All the deaths in our analyses were classified as CRC-specific or non-cancer-related. The time between the date of CRC diagnosis and the date of CRC death is used to calculate the CSS of the CRC-specific death. The expiration date for non-CRC related death is the date of death due to causes other than CRC. The Kaplan–Meier method was used to present the differential prognostic impact of multiple clinicopathologic factors with the log-rank test. P values less than 0.05 were considered statistically significant. Statistical analysis was performed using the Statistical Package for Social Science (SPSS version 23; IBM Corp, Armonk, NY, USA).

## Results

### Clinicopathological factors and conditional survival of oldest-old patients with CRC

A total of 17,359 oldest-old patients diagnosed with CRC were recruited from the SEER database. The median follow-up time was 25 (range, 0–154) months, which was attributed to the dismal survival of oldest-old patients. Death information was counted at the end of follow-up, and a total of 4352 (25.1%) patients died of CRC. The clinicopathologic factors of the entire cohort were summarized in Table 1. Among the oldest-old patients with CRC, parameters including black race, female sex, distal colon and rectum, higher tumor grade, mucinous adenocarcinoma, signet ring cell carcinoma and higher AJCC TNM stage were more likely to be associated with elevated CEA levels (*P* < 0.001). In addition, patients with elevated CEA levels were more likely to receive chemotherapy (12.6% VS. 8.5%, *P* < 0.001).

**Table 1:** Baseline characteristics of oldest old diagnosed with colorectal cancer

Characteristics	No. (%)		*P* value
	**Normal CEA (Stage C0)** **(*N*** **= 9238)**	**Elevated CEA (Stage C1)** **(*N*** **= 8121)**	
**Race**			< 0.001
White	8210 (88.9)	6846 (84.3)	
Black	501 (5.4)	599 (7.4)	
Other	527 (5.7)	676 (8.3)	
**Gender**			< 0.001
Male	3809 (41.2)	2875 (35.4)	
Female	5429 (58.8)	5246 (64.6)	
**Tumor location**			< 0.001
Cecum	2381 (25.8)	2025 (24.9)	
Ascending colon	2096 (22.7)	1613 (19.9)	
Hepatic flexure	475 (5.1)	446 (5.5)	
Transverse colon	925 (10.0)	842 (10.4)	
Splenic flexure	260 (2.8)	210 (2.6)	
Descending colon	346 (3.7)	315 (3.9)	
Sigmoid Colon	1231 (13.3)	1236 (15.2)	
Rectosigmoid junction	421 (4.6)	380 (4.7)	
Rectum	1103 (11.9)	1054 (13.0)	
**Tumor grade**			< 0.001
Grade I	707 (7.7)	489 (6.0)	
Grade II	6246 (67.6)	5167 (63.6)	
Grade III	1704 (18.4)	1668 (20.5)	
Grade IV	214 (2.3)	233 (2.9)	
Unknown	367 (4.0)	564 (6.9)	
**Histology**			< 0.001
Adenocarcinoma	8508 (92.1)	7173 (88.3)	
Mucinous adenocarcinoma	673 (7.3)	829 (10.2)	
Signet-ring cell carcinoma	57 (0.6)	119 (1.5)	
**7th AJCC TNM stage**			< 0.001
I	2736 (29.6)	1280 (15.8)	
IIA	3318 (35.9)	2543 (31.3)	
IIB	348 (3.8)	417 (5.1)	
IIC	95 (1.0)	138 (1.7)	
IIIA	274 (3.0)	164 (2.0)	
IIIB	1556 (16.8)	1564 (19.3)	
IIIC	614 (6.6)	812 (10.0)	
IVA	181 (2.0)	661 (8.1)	
IVB	116 (1.3)	542 (6.7)	
**Chemotherapy**			< 0.001
No/unknown	8453 (91.5)	7101 (87.4)	
Yes	785 (8.5)	1020 (12.6)	

**Fig. 2:**
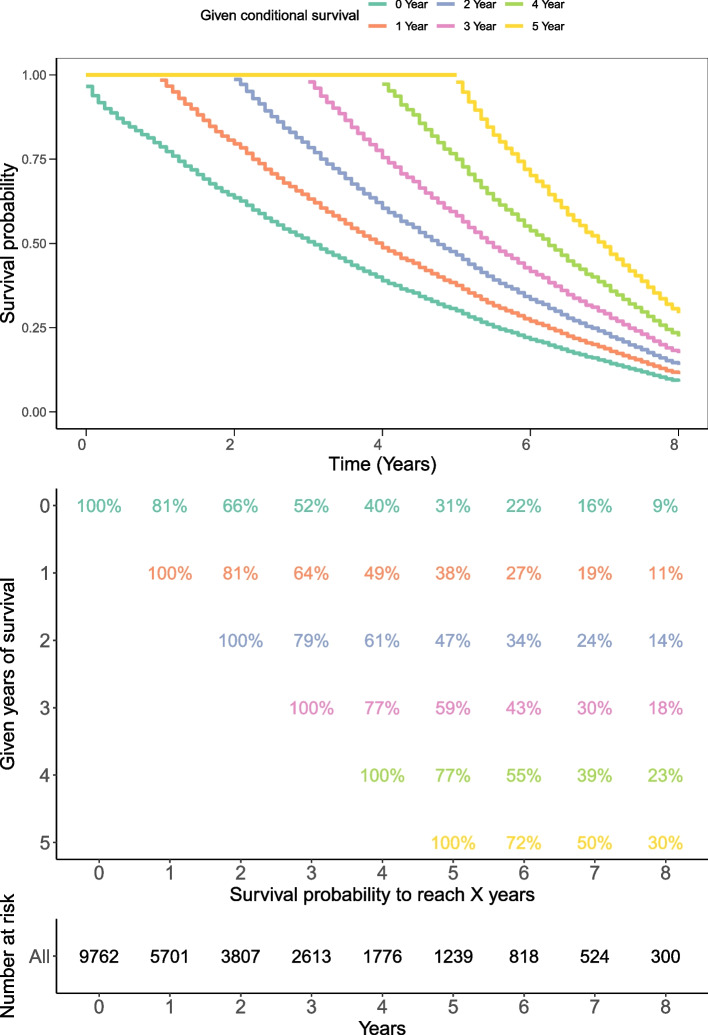
Kaplan–Meier estimates for conditional survival up to 8 years in oldest-old patients with CRC

The conditional survival of oldest-old patients with CRC was illustrated in Fig. 2. The probability of survival increased with each year. Patients who still survived were estimated relative to the total survival time. It should be noted that the 5-year postoperative survival rate increased from 31% after direct surgery to 38%, 47%, 59% and 77% per additional year of survival. The 1-year conditional survival decreased from 81% directly to 77% and 72% at 3 and 5 years. The prognosis of oldest-old patients with CRC was dismal, and the 1-year conditional survival was decreased even after five years with regard to the total years of survival. The 1-year conditional survival of oldest-old patients with CRC was only 11%, 18% and 30% in terms of 1, 3 and 5-year survival.

### C stage is a strong prognostic factor

As shown in Table 2, the characteristics of patients with a *P* value less than 0.20, derived from univariate Cox analyses, were involved in multivariate Cox analyses. The latter showed that the variables including race, gender, tumor location, tumor grade, histology, AJCC stage and receipt of chemotherapy were independent prognostic factors in oldest-old patients with CRC (*P* < 0.001). More importantly, patients with stage C1 had a 48.5% increased risk of CRC-specific mortality (HR = 1.485, 95%CI = 1.393–1.583, using stage C0 patients as reference, *P* < 0.001). In addition, C stage was still an independent prognostic factor in oldest-old patients with non-metastatic CRC and stage C1 was independently associated with 49.8% increased risk of CRC-specific mortality compared with stage C0 (HR = 1.498, 95%CI = 1.399–1.605, using stage C0 as reference, *P* < 0.001; Table 3).

**Table 2:** Univariate and multivariable Cox regression analyses of clinicopathological factors in oldest old diagnosed with colorectal cancer

**Groups**		**Univariate analyses**		**Multivariate analyses**	
	**Variable**	**HR (95%CI)**	***P***	**HR (95%CI)**	***P***
**C-stage**			<0.001		<0.001
	**Stage C0**	Reference		Reference	
	**Stage C1**	2.052 (1.931–2.180)		1.485 (1.393–1.583)	
**Race**			<0.001		0.007
	**White**	Reference		Reference	
	**Black**	1.322 (1.181–1.481)		1.200 (1.071–1.345)	
	**Other**	1.133 (1.013–1.266)		0.994 (0.889–1.112)	
**Gender**			0.154		0.142
	**Male**	Reference		Reference	
	**Female**	1.046 (0.983–1.113)		1.049 (0.984–1.117)	
**Tumor location**			<0.001		<0.001
	**Cecum**	Reference		Reference	
	**Ascending colon**	0.807 (0.737–0.884)		0.884 (0.807–0.968)	
	**Hepatic flexure**	0.868 (0.749–1.006)		0.963 (0.830–1.116)	
	**Transverse colon**	0.798 (0.708–0.889)		0.874 (0.775–0.985)	
	**Splenic flexure**	0.983 (0.812–1.190)		0.959 (0.792–1.161)	
	**Descending colon**	0.866 (0.727–1.031)		0.942 (0.791–1.123)	
	**Sigmoid Colon**	1.099 (0.999–1.209)		1.121 (1.018–1.235)	
	**Rectosigmoid junction**	1.332 (1.164–1.525)		1.324 (1.155–1.516)	
	**Rectum**	1.303 (1.184–1.433)		1.443 (1.307–1.593)	
**Tumor grade**			<0.001		<0.001
	**Grade I**	Reference		Reference	
	**Grade II**	1.145 (1.003–1.308)		1.011 (0.885–1.154)	
	**Grade III**	1.718 (1.492–1.979)		1.248 (1.081–1.441)	
	**Grade IV**	2.157 (1.763–2.638)		1.445 (1.179–1.772)	
	**Unknown**	3.132 (2.667–3.679)		1.641 (1.390–1.937)	
**Histology**			<0.001		0.003
	**Adenocarcinoma**	Reference		Reference	
	**Mucinous adenocarcinoma**	0.915 (0.858–0.975)		0.829 (0.741–0.928)	
	**Signet-ring cell carcinoma**	1.645 (1.427–1.896)		0.862 (0.667–1.115)	
**7th AJCC TNM stage**			<0.001		<0.001
	**I**	Reference		Reference	
	**IIA**	1.059 (0.955–1.175)		1.076 (0.968–1.195)	
	**IIB**	2.440 (2.091–2.846)		2.341 (2.002–2.737)	
	**IIC**	4.220 (3.375–5.276)		3.862 (3.082–4.840)	
	**IIIA**	1.294 (1.021–1.642)		1.339 (1.055–1.700)	
	**IIIB**	2.418 (2.181–2.681)		2.463 (2.214–2.740)	
	**IIIC**	4.235 (3.781–4.744)		4.066 (3.611–4.578)	
	**IVA**	8.991 (7.942–10.179)		8.119 (7.120–9.258)	
	**IVB**	12.738 (11.198–14.488)		10.873 (9.459–12.500)	
**Chemotherapy**			<0.001		<0.001
	**No/unknown**	Reference		Reference	
	**Yes**	1.474 (1.355–1.604)		0.662 (0.604–0.724)

**Table 3:** Univariate and multivariable Cox regression analyses of all clinicopathological factors in oldest old diagnosed with non-metastatic colorectal cancer

**Groups**		**Univariate analyses**		**Multivariate analyses**	
	**Variable**	**HR (95%CI)**	***P***	**HR (95%CI)**	***P***
**C-stage**			<0.001		<0.001
	**Stage C0**	Reference		Reference	
	**Stage C1**	1.740 (1.627–1.860)		1.498 (1.399–1.605)	
**Race**			0.001		0.029
	**White**	Reference		Reference	
	**Black**	1.250 (1.096–1.425)		1.195 (1.048–1.363)	
	**Other**	1.142 (1.009–1.294)		1.014 (0.895–1.150)	
**Gender**			0.075		0.839
	**Male**	Reference		Reference	
	**Female**	1.066 (0.994–1.143)		1.007 (0.938–1.082)	
**Tumor location**			<0.001		<0.001
	**Cecum**	Reference		Reference	
	**Ascending colon**	0.830 (0.750–0.919)		0.885 (0.799–0.980)	
	**Hepatic flexure**	0.880 (0.745–1.038)		0.965 (0.817–1.140)	
	**Transverse colon**	0.836 (0.732–0.954)		0.858 (0.752–0.980)	
	**Splenic flexure**	0.990 (0.797–1.230)		1.014 (0.816–1.260)	
	**Descending colon**	0.838 (0.686–1.024)		0.876 (0.717–1.072)	
	**Sigmoid Colon**	1.105 (0.992–1.232)		1.161 (1.041–1.296)	
	**Rectosigmoid junction**	1.352 (1.161–1.575)		1.398 (1.199–1.629)	
	**Rectum**	1.422 (1.279–1.580)		1.607 (1.437–1.797)	
**Tumor grade**			<0.001		<0.001
	**Grade I**	Reference		Reference	
	**Grade II**	1.123 (0.973–1.297)		0.977 (0.846–1.129)	
	**Grade III**	1.700 (1.458–1.981)		1.240 (1.060–1.450)	
	**Grade IV**	2.081 (1.662–2.606)		1.496 (1.191–1.879)	
	**Unknown**	2.215 (1.820–2.697)		1.885 (1.545–2.300)	
**Histology**			0.010		0.001
	**Adenocarcinoma**	Reference		Reference	
	**Mucinous adenocarcinoma**	0.901 (0.797–1.019)		0.800 (0.707–0.906)	
	**Signet-ring cell carcinoma**	1.460 (1.080–1.972)		0.840 (0.619–1.140)	
**7th AJCC TNM stage**			<0.001		<0.001
	**I**	Reference		Reference	
	**IIA**	1.060 (0.956–1.176)		1.103 (0.992–1.226)	
	**IIB**	2.434 (2.086–2.839)		2.381 (2.035–2.786)	
	**IIC**	4.182 (3.345–5.229)		3.780 (3.015–4.739)	
	**IIIA**	1.293 (1.020–1.640)		1.352 (1.065–1.716)	
	**IIIB**	2.415 (2.178–2.677)		2.465 (2.212–2.746)	
	**IIIC**	4.213 (3.761–4.720)		4.037 (3.579–4.554)	
**Chemotherapy**			<0.001		0.001
	**No/unknown**	Reference		Reference	
	**Yes**	1.332 (1.201–1.477)		0.749 (0.671–0.837)	

### Prognostic value of C-TNM stage

Following combination with the C stage, each AJCC TNM stage was assigned to stage C0 or stage C1, including I C0, I C1, IIA C0, IIA C1, IIB C0, IIB C1, IICC0, IIC C1, IIIA C0, IIIA C1, IIIB C0, IIIB C1, IIIC C0, IIIC C1, IVA C0, IVA C1, IVB C0 and IVB C1.

**Fig. 3:**
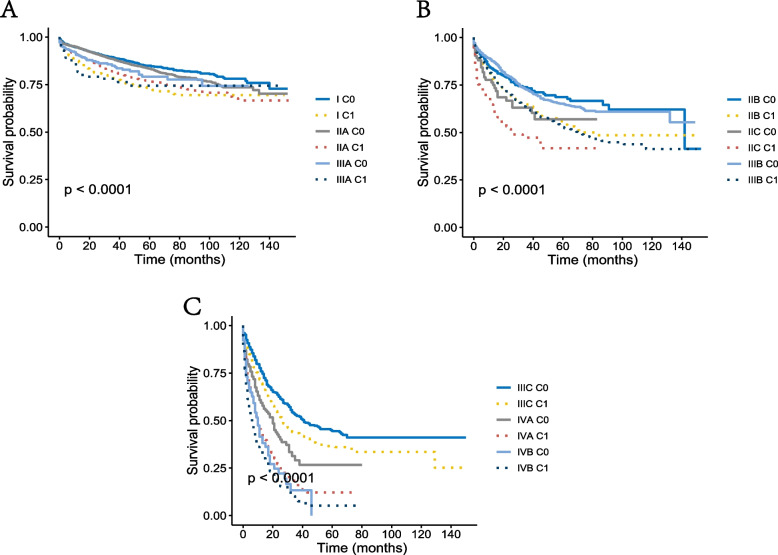
Kaplan–Meier survival curves of C-TNM staging system. **A** Cancer-specific survival (CSS) of I-C 0 stage, I- C 1 stage, IIA-C 0 stage, IIA- C 1 stage, IIIA- C 0 stage, and IIIA- C 1 stage. **B** CSS of IIB- C 0 stage, IIB- C 1 stage, IIC- C 0 stage, IIC- C 1 stage, IIIB- C 0 stage, and IIIB- C 1 stage. **C** CSS of IIIC- C 0 stage, IIIC- C 1 stage, IVA- C 0 stage, IVA- C 1 stage, IVB- C 0 stage, and IVB- C 1 stage

Kaplan–Meier survival analyses showed that in all the respective AJCC TNM stages, CSS was significantly increased in all stage C0 patients compared with stage C1 patients (Fig. 3A-C). Besides, the results were also verified by multivariate Cox analyses. The HR of all stage C0 patients was lower than that of stage C1 patients, which was consistent with Kaplan–Meier survival analysis.

It should also be pointed out that a few patients with stage C1-TNM exceeded stage C0 with higher conventional AJCC TNM stage. For example, the risk of CRC-specific mortality of stage I C1 was significantly higher than that of stage IIA C 0 (HR = 0.626, 95%CI = 0.536–0.731, using stage I C1 as the reference, *P* < 0.001). The risk of CRC specific mortality of stage IIC C1 (HR = 3.408, 95%CI = 2.561–4.534, using stage I C1 as the reference, *P* < 0.001) was higher than that of stage IIIA C0 (HR = 0.773, 95%CI = 0.561–1.065, using stage I C1 as the reference, *P* = 0.115), indicating that stage C1 could upregulate conventional TNM stage (Table 4). In other words, the C stage may have a significant impact on the prognosis of oldest-old CRC patients.

**Table 4:** Prognosis of C-stage and TNM stage in oldest old diagnosed with colorectal cancer

**AJCC TNM staging system**					**TNM-C staging system**				
**Stage**	**Number of the patients**	**Cancer-specific survival**			**Stage**	**Number of the patients**	**Cancer-specific survival**		
		**HR (95% CI)**	**SE**	***P* value**			**HR (95% CI)**	**SE**	***P* value**
**I**	4016	1.00 (Reference)	\	\	**I C0**	2736	0.518 (0.439–0.610)	0.084	< 0.001
					**I C1**	1280	1.00 (Reference)	\	\
**IIA**	5861	1.076 (0.968–1.195)	0.054	0.174	**IIA C0**	3318	0.626 (0.536–0.731)	0.079	<0.001
					**IIA C1**	2543	0.906 (0.776–1.058)	0.079	0.211
**IIB**	765	2.341 (2.002–2.737)	0.080	<0.001	**IIB C0**	348	1.357 (1.061–1.736)	0.126	0.015
					**IIB C1**	417	1.986 (1.611–2.448)	0.107	<0.001
**IIC**	233	3.862 (3.082–4.840)	0.115	<0.001	**IIC C0**	95	2.088 (1.429–3.052)	0.194	< 0.001
					**IIC C1**	138	3.408 (2.561–4.534)	0.146	<0.001
**IIIA**	438	1.339 (1.055–1.700)	0.122	0.016	**IIIA C0**	274	0.773(0.561–1.065)	0.164	0.115
					**IIIA C1**	164	1.136 (0.790–1.635)	0.186	0.492
**IIIB**	3120	2.463 (2.214–2.740)	0.054	<0.001	**IIIB C0**	1556	1.408 (1.201–1.651)	0.081	<0.001
					**IIIB C1**	1564	2.111 (1.813–2.457)	0.078	<0.001
**IIIC**	1426	4.066 (3.611–4.578)	0.061	<0.001	**IIIC C0**	614	2.559 (2.141–3.508)	0.091	<0.001
					**IIIC C1**	812	3.277 (2.782–3.861)	0.084	<0.001
**IVA**	842	8.119 (7.120–9.258)	0.067	<0.001	**IVA C0**	181	4.812 (3.769–6.145)	0.125	<0.001
					**IVA C1**	661	6.891 (5.846–8.125)	0.084	<0.001
**IVB**	658	10.873 (9.459–12.500)	0.071	<0.001	**IVB C0**	116	7.292 (5.548–9.584)	0.139	<0.001
					**IVB C1**	542	9.048 (7.634–10.723)	0.087	<0.001

## Discussion

In the present study, the dismal conditional survival of oldest-old subjects with CRC was carefully considered. The 1-year conditional survival of these patients decreased even after 5 years in terms of overall survival and the 1-year conditional survival of oldest-old patients with CRC was only 11%, 18% and 30% corresponding to 1, 3 and 5 years of survival, respectively.

A previous study [13] demonstrated that elderly patients with CRC exhibited worse overall survival and conditional survival compared with that of young patients with CRC. An additional study [14] examined the conditional survival of long-term CRC survivors in the Netherlands and reported that mortality rates increased with age. The absolute risk of succumbing to CRC ranged from 6.8% to 40.2% depending on age and stage from 0 to 5 years after diagnosis with an increased absolute risk noted with increasing age. Furthermore, patients aged more than 80 years with CRC exhibited a worse conditional 5-year survival (less than50%), which was consistent with the present study [15].

Several factors have been proposed to explain the poor prognosis of the oldest-old patients. The most direct factor is that these patients are more likely to exhibit comorbidities, such as acute renal failure, respiratory failure, cardiac complications, urinary tract infections and pneumonia, which ultimately increases the risk associated with treatment. Accordingly, intensive treatment, such as therapeutic surgery and adjuvant therapy, is less likely to be recommended for elderlypatients [16]. Even after surgical treatment, 17% of patients older than 80 years would develop major complications and 29% of them would experience prolonged length of stay (LOS). The 30-day operative mortality (> 80 years vs. 45–55 years; 6% vs. < 1%), major complications and long-term LOS after laparotomy and laparoscopy were also higher in elderly patients [17]. In addition, the oldest-old CRC patients who did not receive chemotherapy were significantly associated with an increased risk of CRC-related death. This was independent of other prognostic factors in people aged 80 years or older [18]. Moreover, aging itself may reduce physiologic recovery.

The Dukes staging system is the first prognostic system in coloproctology. It is very widely used throughout the world [19]. In the past two decades, CRC has been anatomically staged according to the TNM system, which is based on the anatomic extent of primary tumor (T-stage), lymph node status (N-stage) and the distant spread or metastases (M-stage) classification TNM system by AJCC, [20]. The TNM classification system can project survival estimates by stage. However, the prognostic value of the TNM system is suboptimal in some respects. For example, it would oversimplify the assessment of the biological potential of tumors and the overall risk of recurrence and death [21]. The TNM system requires complete information on CRC patients in addition to Tumor, Node and Metastasis data. Collection of staging data through population-based cancer registries remains a challenge, particularly in low- and middle-income countries. The lack of this information makes it extremely difficult to predict prognosis and affects the choice of treatment for patients [22]. The novel staging system by Sugimoto et al. [23] outperformed the TNM system in predicting survival in stage III colon cancer. These studies may alert the clinicians to the need for more aggressive treatment strategies in patients with early TNM staging and one or more risk factors. It is also important not to ignore the already established risk factors that were not considered in the current TNM staging system. Unfortunately, the oldest-old patients diagnosed with CRC have several risk factors. However, these risk factors cannot be considered by the TNM system, which will lead to overtreatment or undertreatment and affect the prognosis of the disease.

Serum carcinoembryonic antigen (CEA) is a highly glycosylated antigen of 201 kDa, which is expressed on the apical surface of colon epithelial cells and excreted through the intestinal cavity. With the destruction of normal tissue structure in malignant tumors and the loss of polarization of neoplastic cells in the depth of tumor glandular tissue, CEA may be expressed on the entire cell surface and may eventually enter the bloodstream, resulting in an increase in its serum level [24]. The serum level of CEA is a significant tumor marker used to aid in the management of CRC, notably in the preoperative and postoperative assessment of the patients. A higher preoperative CEA level has been identified as an independent and practical predictor of both overall and disease-free survival of CRC [25]. Periodic measurement of CEA levels is important as it could not only reflect the remaining disease when measured postoperatively but also predict cancer recurrence when measured during the surveillance period [26]. A recent study [27] was performed to assess the positive role of CEA in the management of CRC. The data indicated that the 5-year OS and DFS rates for patients with CEA levels ≥ 2.5 ng/ml were 73% and 79% respectively, which were lower than those with CEA level < 2.5 ng/ml (85% and 86% respectively). Recent study has shown that CEA also has a great role in predicting metastatic CRC (mCRC) [28]. In mCRC patients with baseline CEA ≥ 10 ng/ml, CEA levels can predict disease progression after first-line induction therapy in mCRC patients with baseline CEA ≥ 10 ng/ml. A prospective study has noted that CEA kinetic measurements are clinically relevant to the early prediction of treatment outcome in patients with mCRC [29]. When CEA is combined with CA19-9 and CA72-4 to form a joint prediction model, it is more effective in diagnosing and predicting prognosis of CRC patients [8, 30]. Under the circumstances of COVID-19, We believe that it is inconvenient for the elderly to go to the hospital for regular check-ups, especially for some CT and MRI examinations. In this circumstance, by using a liquid biopsy of tumor marker CEA, the prognosis of the elderly can be briefly estimated according to the CEA level. Obviously, as a classical tumor marker, we should spare no effort to explore more value of CEA in clinical diagnosis and treatment, and provide guidance and reference for various CRC patients.

The present study found that stage C1 was independently associated with 48.5% increased risk of CRC-specific mortality compared with stage C0. After combination with C stage, each AJCC TNM stage was divided into stage C0 or stage C1. In all corresponding AJCC TNM stages, the CSS of all stage C0 patients was significantly higher than that of stage C1 patients. It should also be mentioned that a few stage C1-TNM patients exceeded stage C0 and had a higher conventional AJCC TNM stage, suggesting that stage C1 could upregulate conventional TNM stage. To put it differently, the C stage may have a significant impact on the prognosis of oldest-old CRC patients. More importantly, the oldest-old patient group is a specific population in CRC characterized by multiple comorbidities and increased risk of postoperative morbidity and mortality.

The present study contains certain limitations. Initially, the results have to be validated in a larger population sample size. Secondly, detailed patient information is not available in the SEER database. Finally, the analysis is merely based on retrospective data and it is limited by the inherent deficits of its retrospective study nature.

## Conclusion

In conclusion, the dismal conditional survival of oldest-old patients with CRC should be given additional consideration. Furthermore, the study found that stage C1 was independently associated with a 48.5% increased risk of CRC-specific mortality compared with stage C0. All stage C0 patients were associated with significantly increased CSS compared to stage C1 patients across all the respective AJCC TNM stages. It should also be mentioned that the risk of mortality of several stage C1-TNM patients even exceeded stage C0 with higher conventional AJCC TNM stages, indicating that the C stage would influence the prognosis of oldest-old patients with CRC.

## Data Availability

Using the National Cancer Institute’s Surveillance, Epidemiology, and End Results (SEER) *Stat software, Version 8.3.8 (Surveillance Research Program, www.seer.cancer.gov/seerstat), patients meeting the strict criteria were identified from the SEER database.

## Abbreviations

CRC: Colorectal Cancer
HRs: Hazard Ratios
Cis: Confidence Intervals
AJCC: American Joint Committee on Cancer
TNM: Tumor-Node-Metastasis
CEA: Serum carcinoembryonic antigen
C Stage: CEA Levels
CSS: Cancer-specific Survival
LOS: Length of Stay
SEER: The National Cancer Institute’s Surveillance, Epidemiology, and End Results
